# Characterization and genomic analysis of phage vB_ValR_NF, representing a new viral family prevalent in the *Ulva prolifera* blooms

**DOI:** 10.3389/fmicb.2023.1161265

**Published:** 2023-05-05

**Authors:** Xinran Zhang, Yantao Liang, Kaiyang Zheng, Ziyue Wang, Yue Dong, Yundan Liu, Linyi Ren, Hongmin Wang, Ying Han, Andrew McMinn, Yeong Yik Sung, Wen Jye Mok, Li Lian Wong, Jianfeng He, Min Wang

**Affiliations:** ^1^College of Marine Life Sciences, Institute of Evolution and Marine Biodiversity, Key Lab of Polar Oceanography and Global Ocean Change, Frontiers Science Center for Deep Ocean Multispheres and Earth System, Center for Ocean Carbon Neutrality, Ocean University of China, Qingdao, China; ^2^Antarctic Great Wall Ecology National Observation and Research Station, MNR Key Laboratory for Polar Science, Polar Research Institute of China, Shanghai, China; ^3^UMT-OUC Joint Centre for Marine Studies, Qingdao, China; ^4^Institute for Marine and Antarctic Studies, University of Tasmania, Hobart, TAS, Australia; ^5^Institute of Marine Biotechnology, Universiti Malaysia Terengganu, Kuala Terengganu, Malaysia; ^6^College of Environmental Science and Engineering, Tongji University, Shanghai, China; ^7^Haide College, Ocean University of China, Qingdao, China; ^8^The Affiliated Hospital of Qingdao University, Qingdao, China

**Keywords:** *Vibrio phage*, siphovirus, *Ulva prolifera* blooms, genomic and phylogenetic analysis, bacteriophage therapy

## Abstract

**Introduction:**

*Vibrio* is an important bacterial genus containing many pathogenic species. Although more and more *Vibrio* phages were isolated, the genome, ecology and evolution of *Vibrio* phages and their roles in bacteriophage therapy, have not been fully revealed.

**Methods:**

Novel *Vibrio* phage vB_ValR_NF infecting *Vibrio alginolyticus* was isolated from the coastal waters of Qingdao during the *Ulva prolifera* blooms, Characterization and genomic feature of phage vB_ValR_NF has been analysed using phage isolation, sequencing and metagenome method.

**Results and Discussion:**

Phage vB_ValR_NF has a siphoviral morphology (icosahedral head 114±1 nm in diameter; a tail length of 231±1 nm), a short latent period (30 minutes) and a large burst size (113 virions per cell), and the thermal/pH stability study showed that phage vB_ValR_NF was highly tolerant to a range of pHs (4-12) and temperatures (-20 - 45 °C), respectively. Host range analysis suggests that phage vB_ValR_NF not only has a high inhibitory ability against the host strain *V. alginolyticus*, but also can infect 7 other *Vibrio* strains. In addition, the phage vB_ValR_NF has a double-stranded 44, 507 bp DNA genome, with 43.10 % GC content and 75 open reading frames. Three auxiliary metabolic genes associated with aldehyde dehydrogenase, serine/threonine protein phosphatase and calcineurin-like phosphoesterase were predicted, might help the host *V. alginolyticus* occupy the survival advantage, thus improving the survival chance of phage vB_ValR_NF under harsh conditions. This point can be supported by the higher abundance of phage vB_ValR_NF during the *U. prolifera* blooms than in other marine environments. Further phylogenetic and genomic analysis shows that the viral group represented by *Vibrio* phage vB_ValR_NF is different from other well-defined reference viruses, and can be classified into a new family, named *Ruirongviridae*. In general, as a new marine phage infecting *V. alginolyticus*, phage vB_ValR_NF provides basic information for further molecular research on phage–host interactions and evolution, and may unravel a novel insight into changes in the community structure of organisms during the *U. prolifera* blooms. At the same time, its high tolerance to extreme conditions and excellent bactericidal ability will become important reference factors when evaluating the potential of phage vB_ValR_NF in bacteriophage therapy in the future.

## Introduction

*Vibrio* species are heterotrophic bacteria with genetic and metabolic diversity (de Souza Valente and Wan, 2021). They are the natural inhabitants as free-living or related communities in the marine and brackish water environment worldwide, usually accounting for 0.5%–5% of the total number of marine bacteria (Frans et al., 2013; Thompson and Polz, 2014). Several species of *Vibrio* have a wide range of virulence related genes, which are pathogenic for humans and animals. For example, *Vibrio anguillarum* can infect more than 50 kinds of fishes, molluscs and crustaceans (Vasquez et al., 2020). *Vibrio vulnificus* can cause severe wound infection and primary sepsis, which are usually due to humans eating raw seafood or seawater contaminated wounds (López-Pérez et al., 2019). *Vibrio alginolyticus*, the dominant *Vibrio* species in the ocean infect a variety of aquaculture species, such as sea bream, kuruma prawn, shellfish species and so on, it is also a serious pathogen that infects humans (Dong et al., 2020). The characteristics of easier mass reproduction in summer enable *V. alginolyticus* to spread in the food chain through various kinds of contaminated seafood, which eventually leads to the outbreak of human epidemic bacterial diseases (Emam et al., 2019; Liu et al., 2020).

Although *Vibrio* is a great threat to food and public safety even human health, the limited available treatment options for *Vibrio* infection are still antibiotics (Wong et al., 2015). However, the abuse of antibiotics has led to pathogenic bacteria developing resistance to antibiotics, which greatly reduces the therapeutic effect of *Vibrio* infection (Mok et al., 2019). In this context, bacteriophages as biological control agents that are antagonistic to *Vibrio* have appeared on the horizon.

Bacteriophages are viruses that attack bacteria and lyse sensitive bacterial cells (Zhang et al., 2021). Due to their high host specificity, phages are also considered to be an ideal tool to deal with specific pathogens. In fact, several cases of phage therapy successfully treating bacterial infections have been reported recently, including resistant *Acinetobacter baumannii* (Tan et al., 2021; Wu et al., 2021), *Mycobacterium* (Pamela Rodríguez et al., 2020), and *Pseudomonas* (Oechslin et al., 2017; Yang et al., 2021). These spectacular results indicate that bacteriophages have an extraordinary prospect in the treatment of bacterial infection. In addition, phages have also been shown to play an important role in maintaining ecosystem coordination: they occur in high abundance (to 10^8^/mL) in the oceans and top-down control of microbial growth across ecosystems by infecting and lysing their host cells (Zhao et al., 2009; Xu et al., 2018; Bartlau et al., 2021). Some bacteriophages also affect the metabolic activities of the host through horizontal gene transfer (HGT) or the expression of auxiliary metabolism genes (AMGs) generated by their own coding, thus participating in the global geochemical cycle (Rohwer and Thurber, 2009; Lang et al., 2012).

Due to the potential application of *Vibrio* phages in bacteriophage therapy, the genome information of about 400 *Vibrio* phages has been uploaded to GenBank (Liu et al., 2019). Most of them infect pathogenic *Vibrio*, including *V. cholerae*, *V. parahaemolyticus*, and *V. vulnificus.* Such as *Vibrio anguillarum* phage CHOED, isolated from Chilean mussels, has been proved that it can effectively inhibit *V. anguillarum*, thus protect fish from vibriosis in fish farms (Romero et al., 2014). Phage KVP40 has a wide host range, including *V. cholerae, V. parahaemolyticus* and other non-pathogenic species (Miller et al., 2003). Lytic phage pVa-21infecting several fish pathogenic Harveyi clade bacteria, showed potential at biofilm eradication mechanism (Kim et al., 2019). In addition, previous literature has verified the presence of enzymatic functional domains in uncharacterized proteins of many *Vibrio* phages, their roles can be used for the understanding of phage-host interaction and pathogenicity (Sanmukh et al., 2010). Thus, more *Vibrio* phages should be isolated to expand the genetic database of bacteriophages, so as to ensure that *Vibrio* phages can be successfully applied to the antimicrobial treatment of *Vibrio* infection. In this study, a novel *Vibrio* phage vB_ValR_NF, capable of inhibiting *V. alginolyticus* growth was isolated and characterized. This study not only expands the scientific understanding of phage biology, and genomic information, but represents a good model for the exploitation of marine host-phage interactions. The potential usefulness of this phage against *V. alginolyticus*, may provide insight into the best combination of various phages for use in a phage cocktail.

## Materials and methods

### Preparation of bacterial strain

The *V. alginolyticus* strain ATCC 17749 was used as the host for bacteriophage, isolated from the seawater off the coast of Qingdao (36°06′N, 120°34′E) during the *U. prolifera* blooms in July 2019 and were inoculated in liquid Zobell medium at 28°C (Wang Q. et al., 2020).

The genome sequence of the *V. alginolyticus* strain ATCC 17749 is available under the GenBank accession number NZ_BATK00000000. The phylogenetic tree based on 16S rRNA sequence of the *V. alginolyticus* strain ATCC 17749 was constructed with the 16S rRNA sequences of other relative hosts using IQ-TREE (Maximum Likelihood method with 1,000 bootstraps; Supplementary Figure S1).

### Isolation, purification of bacteriophages

The *Vibrio* phage vB_ValR_NF was prepared following the standard virus enrichment and double-layer agar methods (Zhang et al., 2021). Briefly, the host cell (1 mL) was added to the seawater sample (25 mL) collected from the coastal waters of Qingdao where the *U. prolifera* blooms in 2019, incubated overnight at 28°C with 10% liquid Zobell medium, then the mixture was centrifuged at 6,000 × g for 10 min. Supernatants that filtered through 0.22 m–pore-size membranes (Millipore), were concentrated with 100KDa Amincon centrifugal filters (Millipore), then 200 μL concentrated samples were co-cultured with the equivalent host cell, plaques were detected by double-layer plate method. Three isolation rounds were performed by picking single plaques to ensure that phages are purified. Then samples were placed in SM buffer [100 mM NaCl, 81.2 mM MgSO4.7H2O, 50 mM Tris–HCl (pH7.5), 0.01% gelatin] for new plaque assays.

### Transmission electron microscopy

20 μL phage suspension was negatively stained with 2% uranyl acetate, then the morphology of the *Vibrio* phage vB_ValR_NF was examined by energy-filtering transmission electron microscopy (TEM, JEOL Model JEM-1200EX) at 100 kV at NICEM (Liu et al., 2018; Li H. et al., 2019). The average size was taken as the reference diameter by measuring three phage particles in the same field of vision.

### One-step growth assay

The one-step growth curve assay of the *Vibrio* phage vB_ValR_NF was performed following the previous method with some modifications (Middelboe et al., 2010; Wang et al., 2021). Generally, phage suspension (MOI = 0.01) was inoculated into exponentially growing cultures of *V. alginolyticus* strain ATCC 17749 (2.13 × 10^13^ cfu/mL). The mixture was allowed to adsorb for 15 min at 28°C, cells were pelleted by centrifugation (13,000 × g, 1 min), then re-suspended and transferred to 50 mL liquid Zobell medium. Samples were taken at 5 min intervals for 1 h and at 10 min intervals for the following hour, at 30 min intervals in the last hour. Each sample was performed in triplicate, using double-layer agar methods for plaque assays and calculating phage titer to draw a standard curve. Burst size was calculated as the ratio of the final count of liberated phage particles to the initial number of infected host cells at the beginning of the test (Zhang et al., 2020; Liu et al., 2021).

### pH/thermal stability

To gain more information on the biological properties of the Vibrio phage vB_ValR_NF, we tested its pH / thermal stability (Thammatinna et al., 2020). Briefly, 100 μL phage suspension was incubated with 900 μL SM buffer under a range of pH conditions from 3 to 12 at 30°C for 2 h. For thermal stability, phage suspension (pH = 7) was incubated for 2 h at −20°C, 4°C, 25°C, 35°C, 45°C, 55°C, 65°C, and 75°C. Both tests were determined using the double-layer plate method under room conditions (30°C; pH = 7). Each sample was performed in triplicate.

### Host range and host lysis investigation

Twelve *Vibrio* strains and other genus strains were used in this study to detect the host of phage vB_ValR_NF. Briefly, dropping 10 μL of phage suspension (MOI = 0.01) on double-layer agar plates was incubated with bacteria. Repeat the above operation three times for each plate, and then samples were incubated overnight at 28°C, to check whether plaques in the plates (Liu et al., 2018).

In addition, in order to test the ability of phage vB_ValR_NF to kill the host *V. alginolyticus*, an *in vitro* lysis assay was determined: *V. alginolyticus* strain ATCC 17749 in exponential phase was co-cultured with phage suspensions at various MOIs (10^−1^, 10^−2^, 10^−3^, 10^−4^, and 10^−5^). The absorbance (OD600nm) was determined at 2 h intervals for 12 h to reflect the infection trend of the host cells. Bacteria without phage inoculation were used as a control (Lu et al., 2017; Chen et al., 2020). After the *in vitro* lysis assay end, we re-measured the final absorbance (OD600nm) value 24 h after infection at different MOIs, and the SPSS software package (version 12.0) was used for determining statistically significant differences using the Kruskal-Wallis test. Each sample was performed in triplicate.

### Genome sequencing and bioinformatic analysis

Phage DNA was extracted by Virus DNA Kit (OMEGA) and sequenced by the Illumina HiSeq PE150 paired terminal sequence method in Beijing Novogene company (Liu et al., 2021). The data gaps between the remaining contigs were filled using GapCloser and GapFiller (Gong et al., 2017). Open reading frames (ORFs) were predicted by GeneMarkS^1^ and RAST^2^ (Besemer and Borodovsky, 2005; Aziz et al., 2008). The protein function and homology of ORFs were predicted using the BLASTP (http://blast.ncbi.nlm.nih.gov/, E-value ≤10^−3^), Pfam^3^ and HHpred tool (E-value ≤10^−3^), which can fit the results of several databases [NCBI^4^ conserved domain database (CDD), Swiss-Prot^5^ and PDB^6^] (Sayers et al., 2009; Gabler et al., 2020; Mistry et al., 2021). The genome map was performed using the CLC Main Workbench 20 (Tohidi and Javanmard, 2020). tRNAscan-SE^7^ was used to test whether the tRNA sequence is in the phage genome (Lowe and Eddy, 1997). The genome sequence of the phage vB_ValR_NF is available under the GenBank accession number MN812722.1.

### Taxonomic and phylogenetic analysis

In order to explore the position of phage vB_ValR_NF in taxonomy and the homology between phage vB_ValR_NF and other phages, we have done the following work. First, the sequence of phage vB_ValR_NF was searched as a query against the NCBI database through tBLASTx (E-value ≤ 10^−5^; query cover ≥ 50%) and the Integrated Microbial Genomes/Virus (IMG/VR) through BLASTp (E-value ≤ 10^−5^; qurey cover ≥ 50%; Paez-Espino et al., 2017; Roux et al., 2021); Then, the sequence of phage vB_ValR_NF and the screened sequences from IMG/VR and the NCBI database were analyzed together with a total of 4,469 *Caudoviricetes* phages in the NCBI RefSeq database [NCBI Virus (nih.gov)] by vConTACT 2.0, Viral clusters (VCs) were identified using ClusterONE with default parameters, network diagram was visualized by Gephi (Bolduc et al., 2017; Bin Jang et al., 2019). According to the results of vConTACT 2.0, phylogenetic and genome comparison analyses of the *Vibrio* phage vB_ValR_NF and its closest relatives were conducted by viptree^8^ (Nishimura et al., 2017). Meanwhile, we use VIRIDIC to calculate intergenomic similarities between phage vB_ValR_NF and other related phages (Moraru et al., 2020).

### Distribution of the *Vibrio* phage vB_ValR_NF in marine environments

Considering that phage vB_ValR_NF was isolated from the *U. prolifera* blooms, it is necessary to discuss the impact of phage vB_ValR_NF in the marine ecosystem. To this end, we selected 154 viral metagenomes from five viral ecological zones (VEZs) of the Global Ocean Viromes (GOV2.0) data set [Arctic (ARC), Antarctic (ANT), bathy-pelagic (BATHY), temperate and tropical epipelagic (EPI), temperate and tropical mesopelagic (MES)] (Gregory et al., 2019), and supplemented 17 viral metagenomes data sets from the *U. prolifera* blooms in summer 2017 in Qingdao offshore (Bioproject number: PRJNA797266) to see the abundance of phage vB_ValR_NF in the global system. To be more rigorous, we also calculated the abundance of other representative phages like *Pelagibacter* phages (Chen et al., 2019), Cyanophages including *Prochlorococcus* phages and *Synechococcus* phages (Labrie et al., 2013), and other *V. alginolyticus* phages. The relative abundances of these viral genomes calculate by CoverM (v0.3.1; Wang et al., 2021), and the normalized data were log10 transformed based on TPM (transcripts per million).

## Results and discussion

### Morphological and biological properties of phage vB_ValR_NF

**Figure 1:**
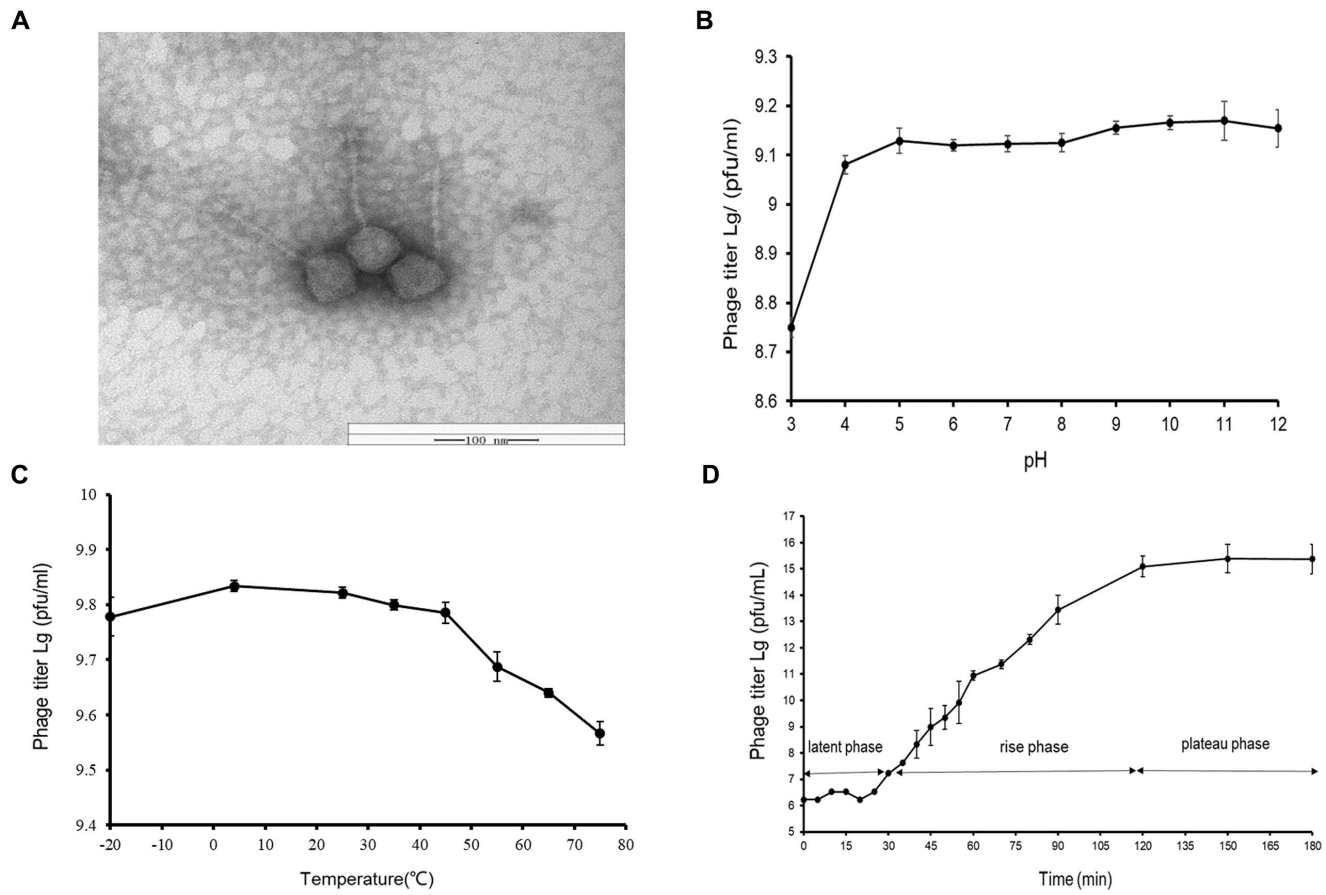
**(A)** Transmission electron micrograph of Vibrio phage vB_ValR_NF. Scale bar, 100 nm. **(B)** pH stability of *Vibrio* phage vB_ValR_NF. Y-axis shows the log of plaque-forming units per milliliter (PFU/mL). **(C)** Temperature stability of *Vibrio* phage vB_ValR_NF. Y-axis shows the log of plaque-forming units per milliliter (PFU/mL). **(D)** One-step growth curve of *Vibrio* phage vB_ValR_NF. Y-axis shows the log of plaque-forming units per milliliter (PFU/mL).

A novel *Vibrio* phage vB_ValR_NF was isolated from the coast of Qingdao during the *Ulva prolifera* blooms. As observed by transmission electron microscope (TEM), the phage vB_ValR_NF has the typical morphology of siphovirus, consisting of an icosahedral capsid (~114 ± 1 nm in diameter) with a long non-contractile tail (~231 ± 1 nm; Figure 1A).

By setting different pH/thermal gradients to treat phage vB_ValR_NF, and using the double-layer plate method under room conditions (30°C; pH = 7), we obtained experimental results on the stability of phage vB_ValR_NF to pH /temperature. As shown in Figure 1B, the phage titer was the lowest at pH 3, subsequently, the phage vB_ValR_NF maintained stable infectivity at the range of pH 4-pH 12, and the highest infectivity to infect the host at pH 11. The thermal stability study showed that phage vB_ValR_NF was able to active between −20°C and 45°C and its infectivity gradually weakens over 45°C, while the phage titer was highest when the temperature was at 5°C (Figure 1C).

The one-step growth curve showed that the latent period of phage vB_ValR_NF was about 30 min and reached the plateau stage after 90 min. The final count of liberated phage particles was 2.42 × 10^15^ PFU/mL (Figure 1D). The burst size was approximately 113 virions per cell. The short latent period and a large burst size indicated that this phage has high lytic activity and robust propagation.

### Host range and host lysis investigation

As shown in Table 1, phage vB_ValR_NF is infectious to several *Vibrio* strains of the *Vibrio* genus. The ability of phages to infect multiple hosts usually means that the genes encoding the tail fibers of phages can recognize specifically related proteins on the surface of many hosts (Ma et al., 2021). In the future, more *Vibrio* strains, especially *Vibrio cholerae* and others related to human diseases, will be used to test the host range of phage vB_ValR_NF. We will also further explore the tail coding genes of phage vB_ValR_NF, better understanding the interaction mechanism between phage and host.

**Table 1:** Host range of *Vibrio* phage NF.

Organism name	Strain	GenBank accession	Total sequence length	Phage NF lysis
*Vibrio atlanticus*	C42	GCA_019845595.1	4,986,964	−
*Vibrio atlanticus*	SM1925	GCA_007786255.1	5,521,369	+
*Vibrio atlanticus*	LGP32	GCA_000091465.1	4,974,818	−
*Vibrio tasmaniensis*	SM1924	GCA_007786305.1	5,791,838	+
*Vibrio cyclitrophicus*	HMSC5	GCA_013113845.1	4,891,316	+
*Vibrio variabilis*	CAIM 1454	GCA_003263785.1	5,082,486	−
*Vibrio variabilis*	T01	GCA_000783325.1	4,529,728	+
*Vibrio variabilis*	JCM 19239	GCA_000755405.1	5,797,527	−
*Vibrio sagamiensis*	NBRC 104589	GCA_000400425.1	4,557,580	+
*Vibrio parahaemolyticus*	2012AW-0154	GCA_009665495.1	5,397,131	+
*Vibrio parahaemolyticus*	20-082E4	GCA_020041945.1	5,621,370	+
*Vibrio parahaemolyticus*	PB1937	GCA_003351885.1	5,580,188	−
*Pseudomonas stutzeri*	GOM4	GCA_019702405.1	4,615,171	−
*Pseudoalteromonas undina*	FME67	GCA_014897915.1	4,237,886	−
*Pseudoalteromonas marina*	ECSMB14103	GCA_002407085.1	3,441,076	−
*Pseudoalteromonas marina*	DSM 17587	GCA_000238335.4	4,190,548	−
*Alteromonas macleodii*	ATCC 27126	GCA_000172635.2	4,653,851	−
*Alteromonas macleodii*	HOT1A3	GCA_001578515.1	4,801,807	−
*Shewanella piezotolerans*	WP3	GCA_000014885.1	5,396,476	−
*Shewanella loihica*	PV-4	GCA_000016065.1	4,602,594	−

**Figure 2:**
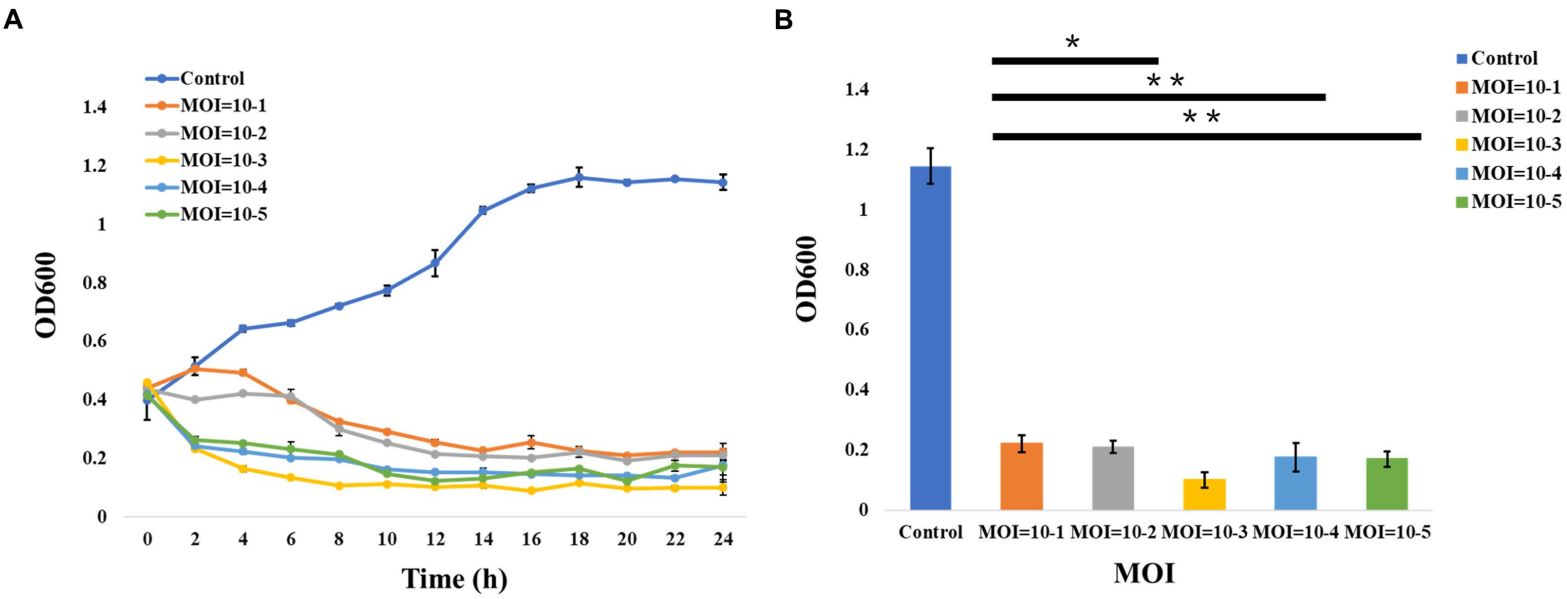
Host lysis investigation. **(A)** The curve of host (*Vibrio alginolyticus* strain) lysis by *Vibrio* phage vB_ValR_NF inoculation at different MOIs (10^−1^, 10^−2^, 10^−3^, 10^−4^, and 10^−5^). **(B)** The final OD600 value after 24 h infection at different MOIs. “*” shows the level of *p*<0.05, “**” shows the level of *p*<0.01, indicating that there is a significant difference between control and the experimental groups. Each point represents average from three replicate experiments.

In addition, the curves of the host lysis test (Figure 2A) indicated that the growth of the bacterial strains was inhibited after phage vB_ValR_NF inoculation at different MOIs, and the phage vB_ValR_NF had the best infection efficiency to the host at the MOI = 10^−3^. The re-determination of OD_600_ value at the end of the host lysis test (Figure 2B) also showed that there was a significant difference (*p* < 0.05) in the number of microorganisms between the experimental groups with phage vB_ValR_NF inoculation and the control group, which indicated that phage vB_ValR_NF had great potential in the treatment of *Vibrio* infection.

### Overall genome features of phage vB_ValR_NF

**Figure 3:**
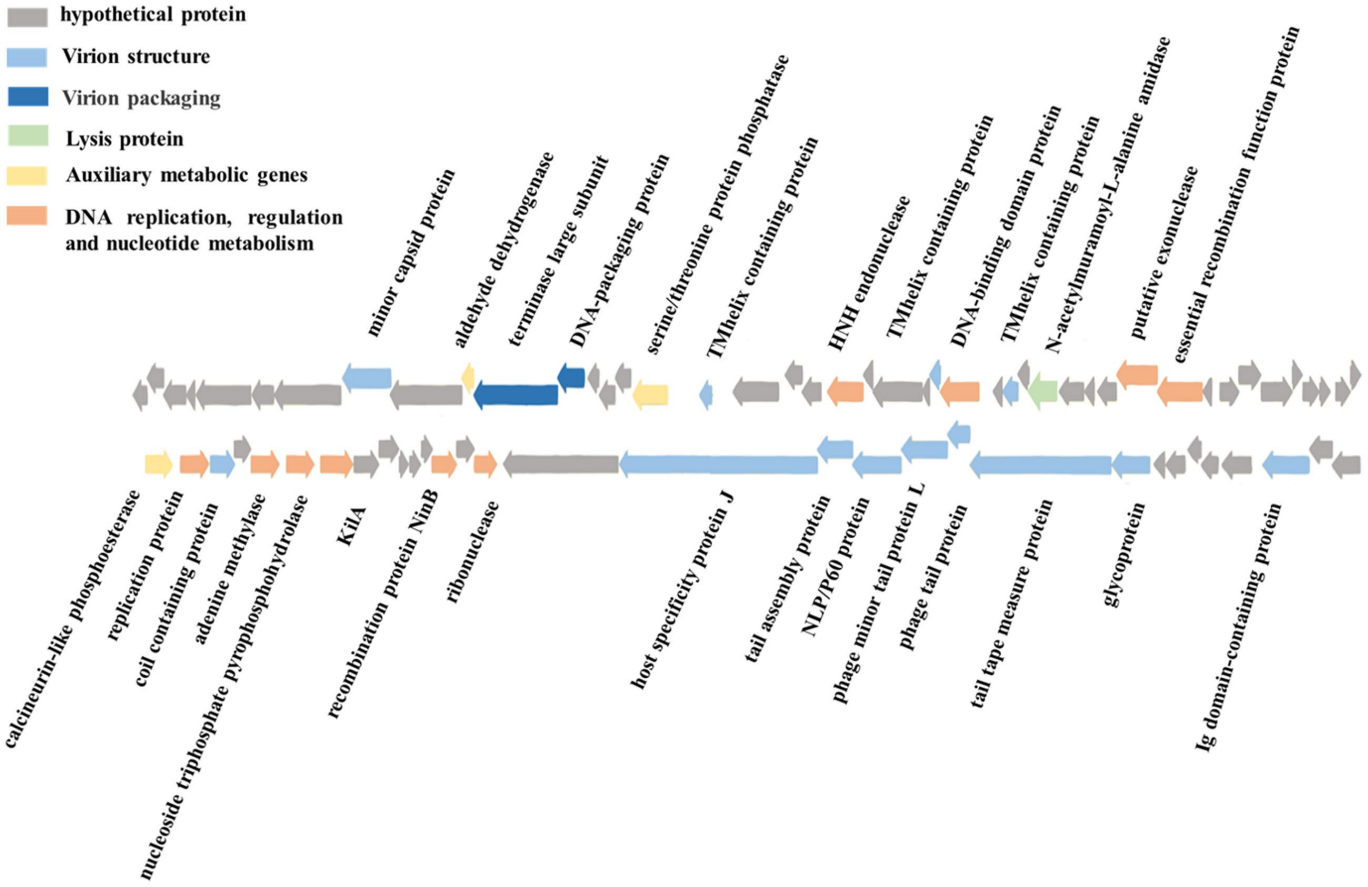
Genome map and functional annotation of the predicted proteins of *Vibrio* phage vB_ValR_NF (gray: hypothetical protein; blue: virion structure module; dark blue: virion packaging module; Green: lysis protein module; Yellow: auxiliary metabolic genes; Orange: DNA replication, regulation and nucleotide metabolism module).

The phage vB_ValR_NF contains a double-stranded DNA genome with a total length of 44,507 bp, 43.10% GC content and there are no tRNAs. Seventy five ORFs were scattered throughout the phage vB_ValR_NF genome with different gene arrangements, among them, the protein functions of 46 ORFs (61%) are unknown, which are called hypothetical proteins. The protein functions of 29 ORFs (39%) can be significantly hit according to the results of searches of the databases, including three auxiliary metabolic genes (AMGs; ORF10, ORF16, ORF 45; Table 2; Figure 3).

**Table 2:** The conserved domains information of *Vibrio* phage NF genome.

No.	Start	Stop	Function	Match phage	E	Accession
8	4,969	4,040	Minor capsid protein	[*Vibrio parahaemolyticus*]	2.00E-144	WP_140079330.1
10	6,552	6,322	Aldehyde dehydrogenase		7.00E-22	TXY57625.1
11	8,162	6,552	Terminase large subunit	[*Vibrio cholerae*]	6.00E-127	AUR98434.1
12	8,667	8,155	DNA-packaging protein		2.00E-64	AUR95850.1
16	10,261	9,599	Serine/threonine protein phosphatase	[*Vibrio* phage 1.251.O._10N.261.55.E5]	2.00E-150	QIW88947.1
17	11,111	10,872	TMhelix containing protein		2.00E-05	AUR83053.1
21	13,318	12,713	HNH endonuclease	[*Vibrio* phage 1.214.O._10N.222.54.F11]	1.00E-104	AUR87732.1
25	14,619	14,434	TMhelix containing protein		1.00E-06	AUR91066.1
26	15,274	14,612	DNA-binding domain protein	[*Vibrio* phage phiV208]	3.00E-33	AUR99418.1
28	15,933	15,667	TMhelix containing protein		3.00E-46	QGF21015.1
30	16,569	16,108	N-acetylmuramoyl-L-alanine amidase	[*Vibrio* phage 1.031.O._10N.261.46.F8]	1.00E-105	QGF21006.1
34	18,283	17,594	Putative exonuclease		2.00E-171	QIW88964.1
35	19,041	18,280	Essential recombination function protein	[*Vibrio* phage 1.103.O._10N.261.52.F2]	4.00E-62	AUR95724.1
45	22,269	22,793	Calcineurin-like phosphoesterase		4.00E-84	AUR92013.1
46	22,958	23,515	Replication protein	[*Vibrio* phage 1.154.O._10N.222.52.B12]	6.00E-22	WP_045357583.1
47	23,544	24,026	Coil containing protein		5.00E-04	AUR92486.1
49	24,339	24,899	Adenine methylase	[*Vibrio* phage 1.265.O._10N.286.52.F6]	1.00E-131	QGF20978.1
50	25,037	25,579	Nucleoside triphosphate pyrophosphohydrolase		3.00E-118	QGF21001.1
51	25,705	26,349	KilA	[*Vibrio* phage Seahorse]	4.00E-141	AUR89334.1
57	27,893	28,381	Recombination protein NinB		1.00E-23	WP_049047713.1
59	28,731	29,171	Ribonuclease	[*Vibrio* phage Seahorse]	6.00E-95	QGF21004.1
61	35,094	31,570	Host specificity protein J		0	WP_150587038.1
62	35,657	35,091	Tail assembly protein	[*Vibrio* phage phiV208]	5.00E-130	QIW88994.1
63	36,423	35,647	NLP/P60 protein		0	QIW88995.1
64	37,157	36,423	Phage minor tail protein L	[*Vibrio* phage 1.211.A._10N.222.52.F11]	6.00E-146	WP_112404072.1
65	37,510	37,154	Phage tail protein		1.00E-58	TNF22126.1
66	39,753	37,510	Tail tape measure protein	[*Vibrio* phage 1.168.O._10N.261.52.A10]	2.00E-75	QGF20992.1
67	40,368	39,763	Glycoprotein		3.00E-145	QIW89000.1
73	42,891	42,154	Ig domain-containing protein	[*Enterobacter hormaechei*]	2.00E-123	QGF21005.1

* indicates *p*<0.05, ** indicates *p*<0.01.

One of the most interesting features of in structure module in phage vB_ValR_NF is the existence of genes for tail assembly protein (ORF 62) and NLP/P60 protein (ORF 63). The tail assembly protein contains a domain that belongs to the family of Lambda_tail_I (PF06805), called bacteriophage lambda tail assembly protein I (TAPI; Iyer et al., 2006; North et al., 2019). TAPI is related to ubiquitin (Ub)-like mediated signaling systems in prokaryotes and a large of phages (Makarova et al., 2000; Li Y. et al., 2019). Similar to the ubiquitin (Ub)-related signal system of eukaryotes, the Ub-like signal system is considered to be widely involved in protein localization, metabolism, function, regulation and degradation (Wu et al., 2018; Valerdi et al., 2021). NlpC/P60 is a ubiquitous cell-wall-associated and papain-like deubiquitinating peptidases (DUBs), it plays an important role in maintaining the dynamic balance between ubiquitination and deubiquitination in eukaryotes, and further participating in the regulation of protein degradation and the regulation of life activities of hosts (Bannantine et al., 2016; Duchêne et al., 2019). Previous works have confirmed many Ub-like proteins in phages and prokaryotes were the precursors to the eukaryotic Ub-signaling system (Iyer et al., 2006; Berk et al., 2020). Consequently, the *Vibrio* phage vB_ValR_NF containing tail assembly protein and NLP/P60 protein may provide an extraordinary model for further studying the evolutionary process of the complete signaling system between viruses, prokaryotes and eukaryotes.

A total of 10 genes were detected to encode proteins related to DNA replication, regulation and nucleotide metabolism, which to some extent explained that the replication system of phage vB_ValR_NF was independent of the host. Interestingly, KilA encoded by ORF 51 is a conserved DNA binding domain, it widely exists in the proteins of large bacteria and eukaryotic DNA viruses (Pritham et al., 2007), considering that the abundance of viruses infected with eukaryotic algae generally increases during the *U. prolifera* blooms, so we speculate that the ORF encoding kilA was detected in phage vB_ValR_NF, which may indicate that there may be complex horizontal gene transfer between microbe during this period (Guo et al., 2011; Jung et al., 2018; Bhatnagar et al., 2020; Wang J. et al., 2020). Meanwhile, ORF 57, coding DNA adenine methylase (Dam), was detected in the genome of phage vB_ValR_NF. Methylases are ubiquitous in prokaryotes and form Restriction-Modification (RM) systems with endonucleases to resist phage infection (Nathans and Smith, 1975). However, about 20% of phages have coding genes related to methylase. They have been proven to help phages avoid the crisis removed by the RM system in hosts and improve their infection efficiency (Pleška and Guet, 2017). In addition, Dams were found to be essential for the virulence of some members of the *Vibrio*, like *Vibrio cholerae*. Dams overproduction can lead to the attenuation of virulence in *Vibrio*, and then induce protective an immune response in the organism infected by *Vibrio* (Murphy et al., 2013; Kalatzis et al., 2017). We may be able to use phage vB_ ValR_ NF to manipulate the virulence of the host *V. alginolyticus* by encoding Dam, then stimulate the immune defense mechanism of the infected body to resist the attack of *Vibrio*. Therefore, the interaction mechanism between phage vB_ValR_NF and the host will also be the focus of our future research.

Most strikingly, three AMGs were found in the genome of phage vB_ValR_NF, including aldehyde dehydrogenase (ORF 10), serine/threonine protein phosphatase (ORF 16), calcineurin-like phosphoesterase (ORF 45).

*Vibrio alginolyticus* produces many aldehydes in its life cycle. In turn, these toxic substances will cause serious harm to the survival of *V. alginolyticus* itself under the limited living environment (such as the *U. prolifera* blooms; Baburam and Feto, 2021). The aldehyde dehydrogenases (ALDHs) are ubiquitous in all organisms and indispensable intermediate reactants in the biological reaction of toxic aldehyde conversion in non-toxic acid, furthermore, the resulting non-toxic acids mediated by ALDHs are also beneficial for promoting plant growth (Ahvazi et al., 2000; Cho et al., 2021). Therefore, we speculate that phage vB_ValR_NF may help host cells improve the degradation ability of toxic aldehydes by encoding ALDHs to alleviate the energetic and bioconversion bottleneck of hosts during the *U. prolifera* blooms. At the same time, it might maintain the dynamic balance of the micro-ecosystem by indirectly affecting the growth of eukaryotes so as to improve the survival chance of phage vB_ValR_NF under harsh conditions.

Serine/threonine protein phosphatase and calcineurin-like phosphoesterase both catalyze the dephosphorylation of phosphorylated protein molecules to regulate protein expression or protect cells from damage when misfolded proteins are accumulated in the periplasm (Lamp et al., 2013). It is likely that serine/threonine protein phosphatase and calcineurin-like phosphoesterase encoded by phage vB_ValR_NF may catalyze the dephosphorylation process of the hosts and alter hosts activities to allow more effective production of phage DNA or protein under some conditions or hinder the synthesis of some important proteins in the hosts.

The functional proteins found in phage vB_ValR_NF are not only essential to maintain the survival of the phage itself, but also widely involved in the interaction with the host, even having a profound impact on the clinical treatment of *Vibrio* infection. However, most of the proteins encoded by phage vB_ValR_NF are labeled as hypothetical proteins, which is due to the small number of phages isolated and identified, the functions of some important viral proteins cannot be successfully hit in the databases. Therefore, the isolation of more *Vibrio* phages has a profound impact on the supplement of the database related viruses, even the clinical treatment of *Vibrio* infection.

### Phylogenetic analysis and comparative genomes

**Figure 4:**
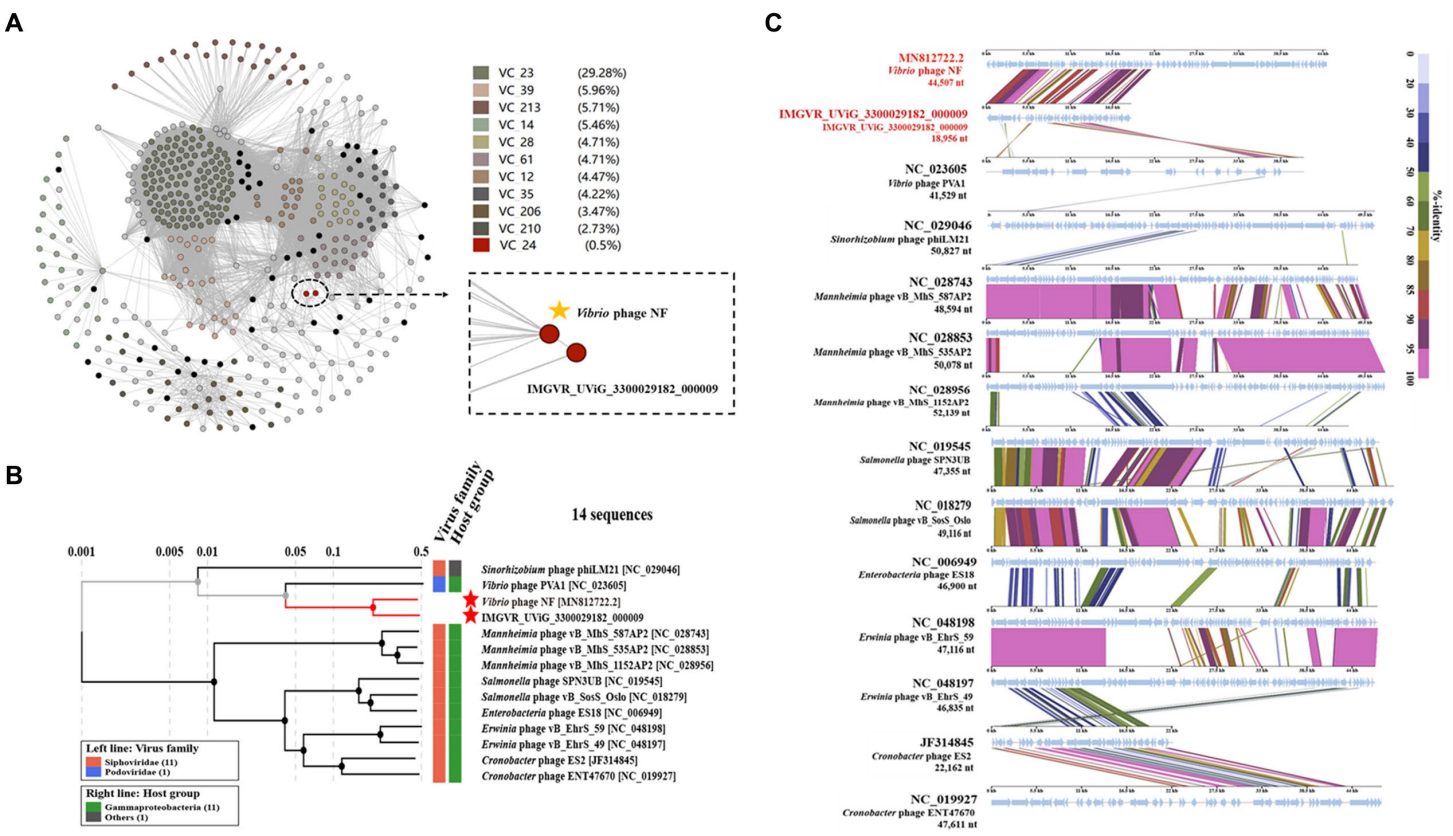
**(A)** Gene-content-based viral network of *Vibrio* phage vB_ValR_NF with related *Caudoviricetes* viruses in the NCBI RefSeq database, and related uncultured viral genome sequences (UViGs) from IMG/VR dataset. The *Vibrio* phage vB_ValR_NF and 1 sequence named IMGVR_UViG_3300029182_000009 belong to the Viral Cluster_24 (VC_24). **(B)** Phylogenetic analysis based on the genome-wide sequence of *Vibrio* phage vB_ValR_NF with other dsDNA viruses in viptree database. **(C)** Genome-wide comparison of *Vibrio* phage vB_ValR_NF and the most related viruses in the phylogenetic tree based on the genome-wide sequence. Genome regions showing similarity were searched using tBLASTX and the matches satisfying length and *E*-value (<10^−5^) cutoffs were indicated by the rectangle according to the color scale on the right.

**Figure 5:**
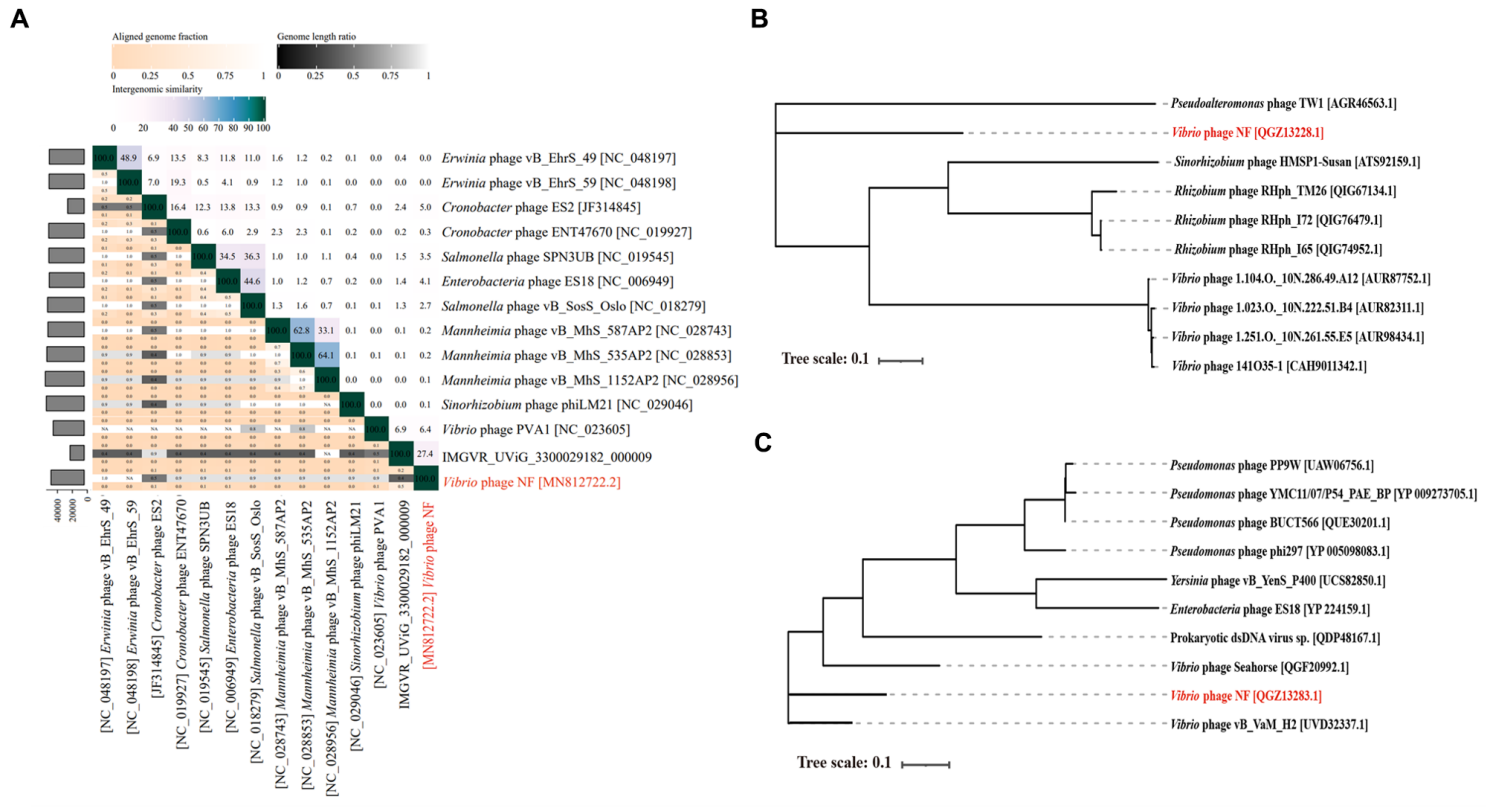
**(A)** The heat map shows nucleotide-based intergenomic similarities of the total of 14 phages from the phylogenetic tree of Figure 4B calculated with VIRIDIC. Values represent Average Nucleotide Identity (ANI). **(B)** The phylogenetic tree is based on terminase large subunits (TerL) of *Vibrio* phage vB_ValR_NF and homolog proteins from other phages. Amino acid sequences were aligned based on the Maximum Likelihood method with 1,000 bootstrap replications by the software IQ-TREE. **(C)** The phylogenetic tree is based on tape measure protein (TMP) of *Vibrio* phage vB_ValR_NF and homolog proteins from other phages. Amino acid sequences were aligned based on the Maximum Likelihood method with 1,000 bootstrap replications by the software IQ-TREE.

Based on search results in the NCBI and the IMG/VR database, only two metagenomic assembled uncultured viral sequences (IMGVR_UViG_3300029182_000002, IMGVR_UViG_3300029182_000009; E-value ≤ 10^−5^; qurey cover ≥ 50%) were matched with phage vB_ValR_NF. Then, vConTACT 2.0 was used to compare the genomic similarity at the protein level between these three sequences and a total of 4,469 *Caudoviricetes* viruses in the NCBI RefSeq database. Only IMGVR_UViG_3300029182_000009 was classified into Viral Cluster 24 (VC_24) together with phage vB_ValR_NF (Figure 4A). The results of phylogenetic (Figure 4B) and genomic comparative analysis (Figure 4C) using viptree also showed that VC_24 is relatively independent of other viral clusters and the level of genes encoding homologous proteins between VC_24 and other phages varies greatly, which may suggest that phage vB_ValR_NF may represent a unique group. To confirm this hypothesis, nucleotide-based intergenomic similarities of the total of 14 phages from the phylogenetic tree of Figure 4B were calculated with VIRIDIC, the result showed that the nucleotide-based intergenomic similarities between vB_ValR_NF and other phages were low (0–27.4%; Figure 5A). Considering that phages are regarded by the ICTV as being members of the same genus when their nucleotide sequence identities are greater than 70% (Moraru et al., 2020), phage vB_ValR_NF is likely to represent a new family under the *Caudoviricetes* viruses. In order to fully verify the reliability of the conclusions, we also used IQ-TREE to build a phylogenetic tree based on the conservative proteins including the TerL (ORF 11) and TMP (ORF 66) of phage vB_ValR_NF with the most matching 10 phage proteins, respectively. The results indicated that phage vB_ValR_NF has conservative proteins distinguished from other phages and form a novel sub-branch in the phylogenetic tree (Figures 5B,C). Therefore, we can propose that phage vB_ValR_NF represents a new family within *Caudoviricetes*, named here as *Ruirongviridae*.

### Distribution of the *Vibrio* phage vB_ValR_NF in marine environments

**Figure 6:**
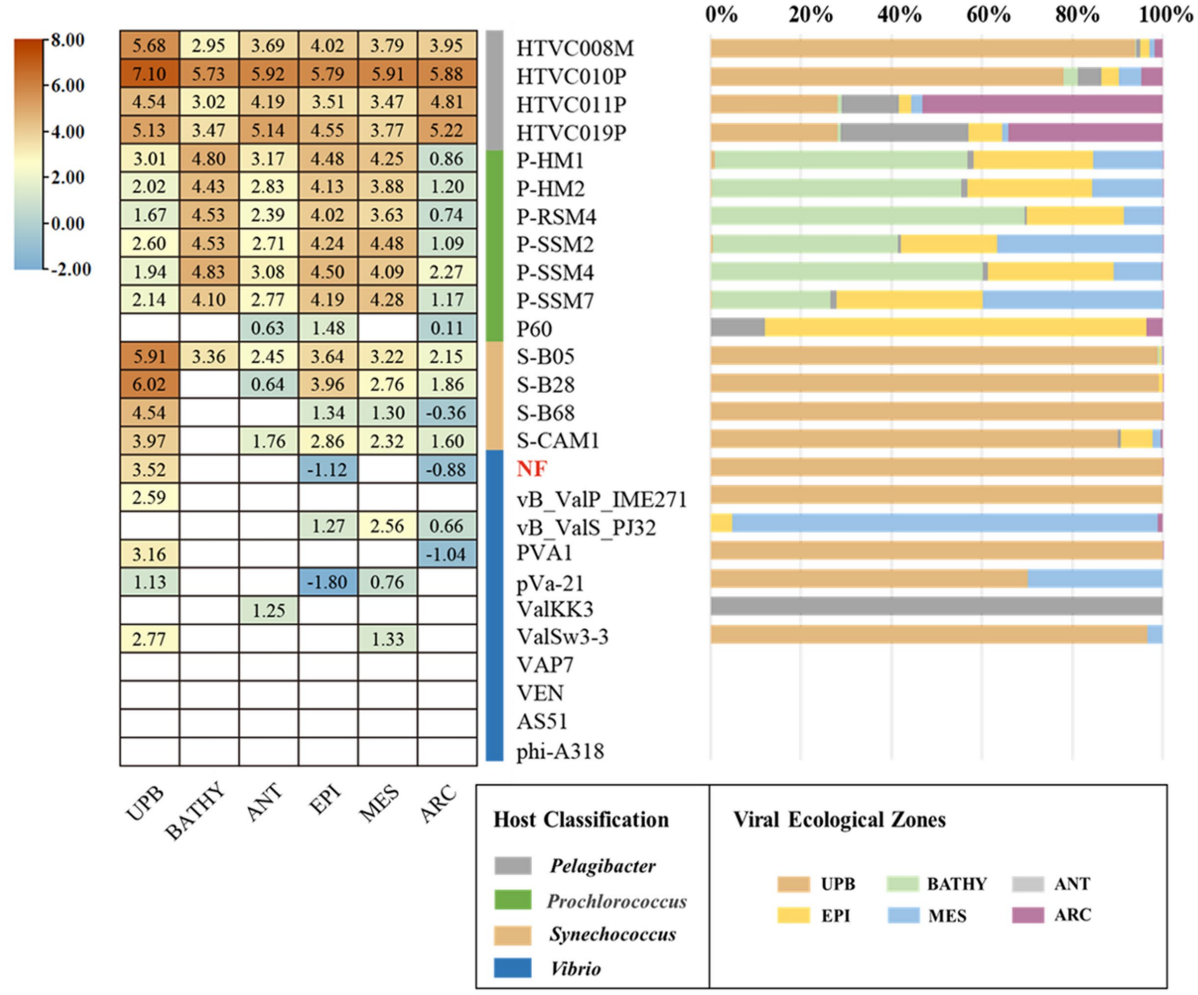
Relative abundance of the *Vibrio* phage vB_ValR_NF in marine environments. Left, the heatmap reflects the relative abundances of the Vibrio phage vB_ValR_NF and other bacteriophages in five marine viral ecological zones (VEZs) defined by the GOV2.0. (ARC, Arctic; ANT, Antarctic; BATHY, bathypelagic; EPI, temperate and tropical epipelagic; MES, temperate and tropical mesopelagic.) and 2017 viral metagenomes data sets from the *Ulva prolifera* blooms in the summer of 2017 in Qingdao offshore (UPB). The normalized data were log10 transformed based on TPM (transcripts per million). Right, the bar graph shows the percentage of bacteriophages in the five VEZs and UPB.

As expected, the relative abundance of *Vibrio* phage vB_ValR_NF is high in the area where the *U. prolifera* blooms off the coast of Qingdao, far exceeding the proportion in these data sets of global ocean viromes (GOV2.0; Figure 6). We also noticed that some other phages, such as *Synechococcus* phage, *Prochlorococcus* phage, etc., were also active during the outbreak of the *U. prolifera*, which also confirmed that viruses might be played a pivotal role in the process of the green tide. Although the microbial community structure during the *U. prolifera* blooms has been studied to some extent (Lin et al., 2017; Wang et al., 2017; Fan et al., 2022), there is no report on the single phage isolated during this period. Therefore, *Vibrio* phage vB_ValR_NF will be an ideal model to help us understand how a single virus affects the host, the microbial loop, and even the whole ecosystem. At present, we have only compared the *Vibrio* phage vB_ValR_NF with viral metagenomes data sets from the *U. prolifera* blooms in the summer of 2017 in Qingdao offshore, which is not enough to help us further study the impact of *Vibrio* phage vB_ValR_NF on Qingdao offshore environment. Therefore, we will also collect more viral metagenomes data sets during the *U. prolifera blooms* in the future for a more in-depth discussion on the functions of phage vB_ValR_NF.

## Conclusion

In this study, *Vibrio* phage vB_ValR_NF was isolated from the coastal area of Qingdao, China in 2019 (36°06′N, 120°34′E), where the *U. prolifera* blooms. Genomics analysis shows that *Vibrio* phage vB_ValR_NF is distanced from other reference viruses in terms of evolutionary relationship, and has unique conservative protein genes far from other phages. We propose that it represents a new family in the *Caudoviricetes* viruses, called *Ruirongviridae*. The stability, growth curve and host range analysis suggest that phage vB_ValR_NF could be active in a high range of pH and temperature, with a short latent period and a large burst size, and has a high inhibitory ability against the host *V. alginolyticus* and other strains of *Vibrio* genus, these characteristics will become important reference factors when evaluating the potential of phage vB_ValR_NF in bacteriophage therapy in the future. In addition, *Vibrio* phage vB_ValR_NF was more abundant during the *U. prolifera* blooms than in other marine environments, the existence of three AMGs and RM modification system also seems to mean that it can promote the host and itself to occupy the survival advantage during the *U. prolifera* blooms by influencing some metabolic mechanisms of the host, suggest it may play a key role in affecting the microbial community structure and the micro-environment during the occurrence and decline of *U. prolifera* blooms. In view of the great potential of *Vibrio* phage vB_ValR_NF in bacteriophage therapy and its important role in the *U. prolifera* blooms ecosystem, we will focus on these aspects to discuss and analyze phage vB_ValR_NF in the future.

## Data availability statement

The datasets presented in this study can be found in online repositories. The names of the repository/repositories and accession number(s) can be found at: https://www.ncbi.nlm.nih.gov/genbank/, MN812722.1.

## Author contributions

YaL, JH, AM, and MW: conceptualization, revision, project administration, supervision, and funding acquisition. XZ, KZ, YuL, YH, and LR: methodology, formal analysis, and writing and original draft preparation. XZ, YD, ZW, and HW: sample collection and expedition organization. YS, WM, and LW: review and editing. All authors contributed to the article and approved the submitted version.

## Funding

This work was supported by the Laoshan Laboratory (no. LSKJ202203201), National Key Research and Development Program of China (2022YFC2807500), Natural Science Foundation of China (nos. 41976117, 42120104006, 42176111, and 42188102), and the Fundamental Research Funds for the Central Universities (202172002, 201812002, and 202072001 to AM).

## Conflict of interest

The authors declare that the research was conducted in the absence of any commercial or financial relationships that could be construed as a potential conflict of interest.

## Publisher’s note

All claims expressed in this article are solely those of the authors and do not necessarily represent those of their affiliated organizations, or those of the publisher, the editors and the reviewers. Any product that may be evaluated in this article, or claim that may be made by its manufacturer, is not guaranteed or endorsed by the publisher.

## Footnotes

^1^http://topaz.gatech.edu/GeneMark/

^2^http://rast.nmpdr.org/

^3^http://pfam.xfam.org/search/sequence

^4^http://www.ncbi.nlm.nih.gov/

^5^https://web.expasy.org/docs/swiss-prot_guideline.html

^6^http://www.rcsb.org/

^7^http://lowelab.ucsc.edu/tRNAscan-SE/

^8^http://www.genome.jp/viptree
