# Supplementary figures and images for: Characterization and genomic analysis of phage vB_ValR_NF, representing a new viral family prevalent in the Ulva prolifera blooms
Source: Front Microbiol. 2023 May 5;14:1161265. doi: 10.3389/fmicb.2023.1161265 (PMC10196503; doi:10.3389/fmicb.2023.1161265)

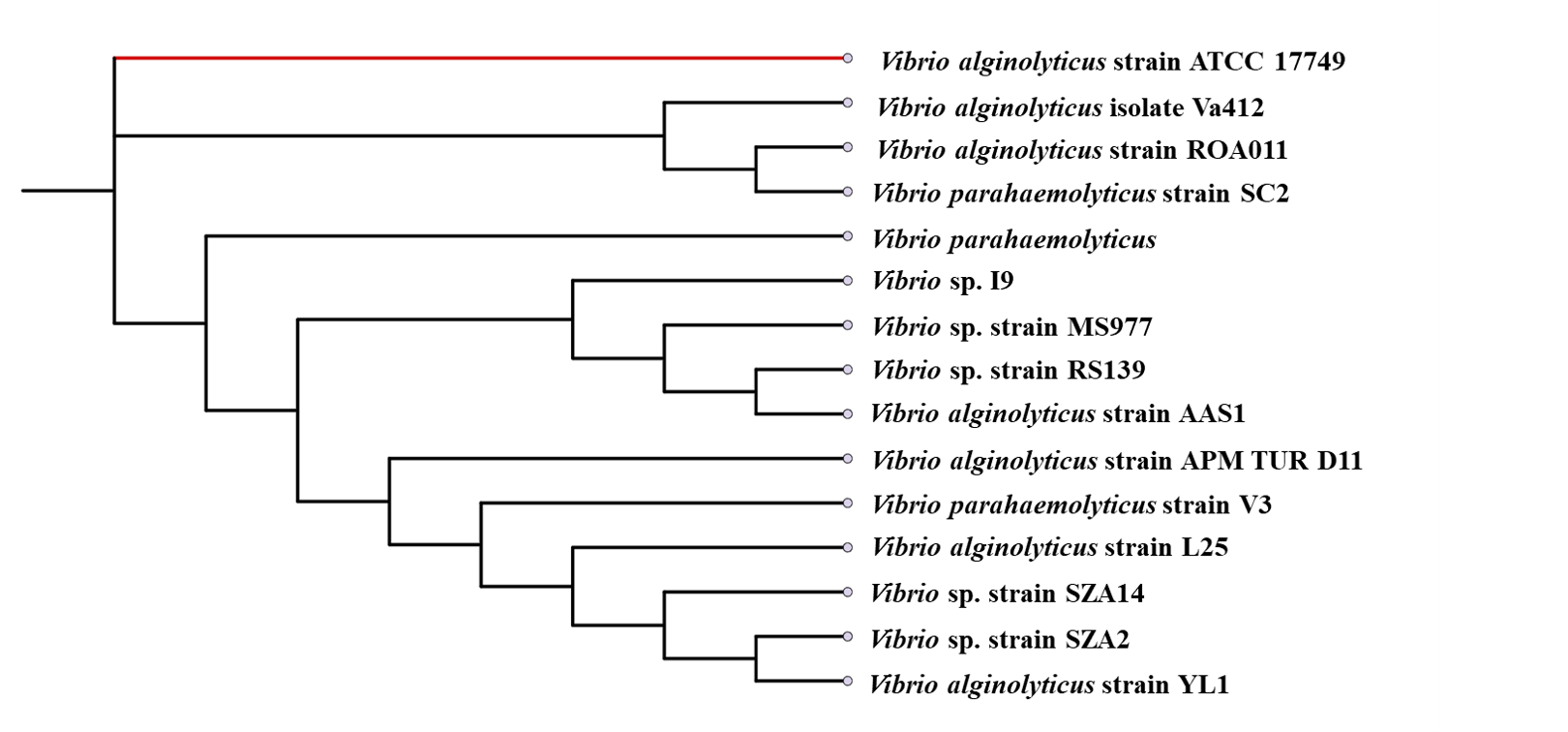

Supplement: Supplementary file 1 [file Image_1.TIF]
